# Amino Acid-Based Diet Prevents Lethal Infectious Diarrhea by Maintaining Body Water Balance in a Murine *Citrobacter rodentium* Infection Model

**DOI:** 10.3390/nu13061896

**Published:** 2021-05-31

**Authors:** Tatsuki Kimizuka, Natsumi Seki, Genki Yamaguchi, Masahiro Akiyama, Seiichiro Higashi, Koji Hase, Yun-Gi Kim

**Affiliations:** 1 Research Center for Drug Discovery, Faculty of Pharmacy and Graduate School of Pharmaceutical Sciences, Keio University, Tokyo 105-8512, Japan; kimizukatatsuki@keio.jp (T.K.); natsumisoshiru826@keio.jp (N.S.); 82137494@keio.jp (G.Y.); akiyama.masahiro@keio.jp (M.A.); 2 Division of Biochemistry, Faculty of Pharmacy and Graduate School of Pharmaceutical Sciences, Keio University, Tokyo 105-8512, Japan; hase-kj@pha.keio.ac.jp; 3 Co-Creation Center, Meiji Holdings Co., Ltd., Tokyo 192-0919, Japan; seiichirou.higashi@meiji.com

**Keywords:** amino acid-based diet, *Citrobacter rodentium*, dehydration, diarrhea, enteric infection

## Abstract

Infectious diarrhea is one of the most important health problems worldwide. Although nutritional status influences the clinical manifestation of various enteric pathogen infections, the effect of diet on enteric infectious diseases remains unclear. Using a fatal infectious diarrheal model, we found that an amino acid-based diet (AD) protected susceptible mice infected with the enteric pathogen *Citrobacter rodentium*. While the mice fed other diets, including a regular diet, were highly susceptible to *C. rodentium* infection, AD-fed mice had an increased survival rate. An AD did not suppress *C. rodentium* colonization or intestinal damage; instead, it prevented diarrhea-induced dehydration by increasing water intake. An AD altered the plasma and fecal amino acid levels and changed the gut microbiota composition. Treatment with glutamate, whose level was increased in the plasma and feces of AD-fed mice, promoted water intake and improved the survival of *C. rodentium*-infected mice. Thus, an AD changes the systemic amino acid balance and protects against lethal infectious diarrhea by maintaining total body water content.

## 1. Introduction

The World Health Organization defines diarrhea as the passage of three or more loose or liquid stools per day (or more frequent passage than is normal for the individual). Acute diarrheal illness is presently one of the most important health problems worldwide, particularly in young children in developing countries. Bacterial infectious diarrhea is the most prevalent diarrheal disease worldwide [1,2,3,4]. Commonly reported enteric bacterial diarrhea and causative agents include *Escherichia coli* gastroenteritis, Salmonellosis (various *Salmonella* serovars), Shigellosis (*Shigella* spp.), Campylobacter gastroenteritis (*Campylobacter jejuni*), cholera (*Vibrio cholerae*), staphylococcal food poisoning (*Staphylococcus aureus* enterotoxins), and botulism (*Clostridium botulinum*) [5,6]. Enteropathogenic and enterohemorrhagic *E. coli* (EPEC and EHEC, respectively) are among the most important bacterial causes of diarrhea worldwide [7,8,9]. These pathogens employ a type-III secretion system (T3SS) and induce ultrastructural changes characterized by intimate bacterial adhesion to the apical surface of enterocytes, microvilli effacement, and pedestal formation, which are called “attaching and effacing” (A/E) lesions [10].

*Citrobacter rodentium*, a mouse pathogen that harbors a homologous T3SS and induces A/E lesions, has been extensively used as a model for studying human EPEC and EHEC infection [11,12]. *C. rodentium* typically causes self-limiting epithelial hyperplasia with a variable degree of inflammation in the distal colon of most laboratory mouse lines [13,14]. In contrast, suckling animals or C3H strains demonstrate severe colonic inflammation, diarrhea, and high mortality after infection with *C. rodentium* [15,16,17].

Nutritional status influences morbidity and mortality in diarrheal and enteric diseases [18,19]. For example, the nutritional management of dietary protein is effective for persistent diarrhea and the reduction of stool output [20,21]. In addition, an increasing number of studies indicate that dietary amino acids play important roles in suppressing intestinal inflammation [22,23]. Furthermore, several dietary factors have been shown to regulate *C. rodentium* colonization and infection-induced intestinal inflammation. Dietary quercetin and chitosan increase gut microbial diversity and attenuate colitis severity in *C. rodentium*-infected mice [24,25]. Diet-derived galacturonic acid suppresses virulence factor expression and inhibits intestinal *C. rodentium* colonization [26]. In contrast, a Western-style diet and insufficient dietary choline induce gut dysbiosis and severe pathology in response to *C. rodentium* infection [27,28]. However, only a few dietary factors that improve mortality in bacterial infectious diarrhea have been identified.

In this study, we identified a diet that can impact mortality in susceptible mice infected with *C. rodentium*.

## 2. Materials and Methods

### 2.1. Mice

Four-week-old female C3H/HeN mice were purchased from CLEA Japan Inc. (Tokyo, Japan). After a 2 week acclimatization period, the mice were given ad libitum access. to one of four diets: regular natural diet (RD; CE2, CLEA Japan Inc.), regular purified diet (PD; AIN93G, Research Diets; New Brunswick, NJ, USA), amino acid-based purified diet (AD; an original diet A07060801, Research Diets), and purified high-fat diet (FD; D12492, Research Diets) (Table 1). All mice were housed at the Keio University Faculty of Pharmacy, Tokyo.

### 2.2. C. rodentium Infection

The kanamycin-resistant WT *C. rodentium* strain DBS120 (pCRP1:: Tn5) was used [29]. For inoculation, bacteria were grown overnight in 50 μg/mL kanamycin-supplemented Luria–Bertani (LB) broth with shaking at 37 °C. Mice were infected by oral gavage with 0.2 mL phosphate-buffered saline (PBS) containing approximately 1 × 10^9^ colony-forming unit (CFU) *C. rodentium*. To determine the bacterial load in the feces or tissues, fecal pellets were collected from individual mice, homogenized in cold PBS, plated at serial dilutions on McConkey agar containing 50 μg/mL Kan, and the CFUs was determined after overnight incubation at 37 °C. The mice were sacrificed at various time points post-infection.

### 2.3. Hematoxylin and Eosin Staining of the Colonic Tissue

Colonic tissue samples were fixed in 10% formalin neutral buffer solution (Mildform 10N, Wako Pure Chemical Industries, Osaka, Japan) overnight. After fixation, the samples were embedded in paraffin and then cut into 3 μm sections. The sections were deparaffinized, rehydrated, and stained with hematoxylin (Agilent Technologies, Inc.; Santa Clara, CA, USA) and eosin (Wako Pure Chemical Industries) and then mounted with Mount-Quick (Daido Sangyo Co., Ltd., Saitama, Japan).

### 2.4. Glutamate Administration

Elix water was used to dissolve sodium L-glutamate monohydrate (1% solution; TCI; Tokyo, Japan) and sterilized through a 0.22 μm filter. PD-fed mice were treated with or without glutamate in drinking water from 10 days prior to *C. rodentium* infection until the end of the study.

### 2.5. Amino Acid Concentration

Plasma and fecal free amino acids were determined using an LC/MS system (TQD, Waters Corporation; Milford, MA, USA), with pre-column 6-aminoquinolyl-N-hydroxysuccinimidyl carbamate derivatization [30]. The analytes were initially deproteinized with 5% trichloroacetic acid. After mixing, the samples were centrifuged at 12,000 rpm for 10 min at 4 °C. The supernatant from each sample was assayed for free amino acids. Data were analyzed using the Waters TargetLinks^TM^ software.

### 2.6. Fecal Lipocalin-2

We measured the fecal lipocalin-2 level as a non-invasive intestinal inflammation biomarker [31]. Mouse fecal pellets were collected in sterile 1.5 mL microcentrifuge tubes, and 100 mg/mL suspensions in sterile 0.1% Tween-20/D-PBS (–) were prepared. Samples were shaken using a vortex mixer at maximum speed for 20 min, followed by centrifugation. The supernatants were assayed for lipocalin-2 using mouse Lipocalin-2/NGAL DuoSet ELISA (R&D Systems; Minneapolis, MN, USA).

### 2.7. Reverse Transcription and Quantitative PCR

Total RNA from mouse tissue was extracted using the PureLink RNA Mini Kit (Thermo Fisher Scientific; Waltham, MA, USA) according to the manufacturer’s instructions. RNA was reverse-transcribed to obtain cDNA using the ReverTra Ace qPCR RT Master Mix with gDNA Remover (TOYOBO; Osaka, Japan). RT-qPCR was performed using StepOnePlus (Thermo Fisher Scientific) with THUNDERBIRD SYBR qPCR Mix (TOYOBO). Oligonucleotide primers were purchased from Integrated DNA Technologies (IA, USA). Primer sequences for *Ctnnb1* and *Mmp7* are listed in Table 2.

### 2.8. Colonic Epithelial Barrier Permeability

Fluorescein isothiocyanate (FITC)-dextran (4 kDa; Sigma-Aldrich; St. Louis, MO, USA) was dissolved in PBS at a concentration of 50 mg/mL. Mice were fasted for 4 h prior to gavage with 60 mg FITC-dextran/100 g body weight. Mice were anesthetized 4 h after gavage, and blood was taken from the heart and centrifuged at 1100× *g* for 15 min at 4 °C. Plasma was collected and the fluorescence at an 485 nm excitation wavelength and 528 nm emission wavelength was quantified.

### 2.9. Plasma IgA ELISA

The plasma samples were subjected to IgA ELISA using a mouse IgA ELISA Quantitation Set (Bethyl Laboratories, Inc.; Montgomery, TX, USA), following the manufacturer’s instructions.

### 2.10. Fecal Pellet DNA Extraction and 16S rRNA Gene Sequencing and Analysis

Bacterial DNA was extracted from the feces using an E.Z.N.A^®^ Stool DNA kit (Omega Bio-Tek, Inc.; Norcross, GA, USA) and purified using magLEAD 12gc (Precision System Science Co., Ltd.; Chiba, Japan). PCR was performed using KAPA HiFi HotStart ReadyMix (Nippon Genetics Co.,Ltd.; Tokyo, Japan) and the primer set (forward: 5′-TCGTCGGCAGCGTCAGATGTGTATAAGAGACAGCCTACGGGNGGCWGCAG-3′, and reverse: 5′-GTCTCGTGGGCTCGGAGATGTGTATAAGAGACAGGACTACHVGGGTATCTAATCC-3′) for the V3–V4 regions of 16S rRNA. The amplicons were purified using AMPure XP (Beckman Coulter; Brea, CA, USA). DNA from each sample was added to different index sequences using the Nextera XT index kit (Illumina; San Diego, CA, USA). Mixed samples were prepared by pooling approximately equal amounts of each amplified DNA and sequenced using Miseq Reagent Kit V3 (600 cycle) and a MiSeq sequencer (Illumina), in accordance with the manufacturer’s instructions.

The sequencing data were analyzed using Qiime2 (version 2020.11) [32]. To trim the primer region from the raw sequences, Cutadapt in the Qiime2 plugin was used (https://doi.org/10.14806/ej.17.1.200, accessed on 27 May 2021). The sequences without primer regions were processed for quality control, paired-end read joining, chimera filtering, and amplicon sequence varient (ASV) table construction using the DADA2 algorithm [33]. For each ASV-representative sequence, BLAST [34] was used to assign the taxonomy based on the SILVA database (version 138) [35]. After randomly sampling 4100 reads using a feature table [36], the compositional data were converted, and diversity analysis was performed.

### 2.11. Statistical Analyses

Statistical analyses were performed using GraphPad Prism software (version 9.0.2; GraphPad Software Inc.; San Diego, CA, USA). Differences between two groups were evaluated using the D’Agostino and Pearson test and F-test followed by the Student’s *t*-test, Welch’s *t*-test, or Mann–Whitney *U* test. Differences were considered significant at *p* < 0.05.

## 3. Results

### 3.1. AD Protects the Mice from Lethal C. rodentium Infection

**Figure 1:**
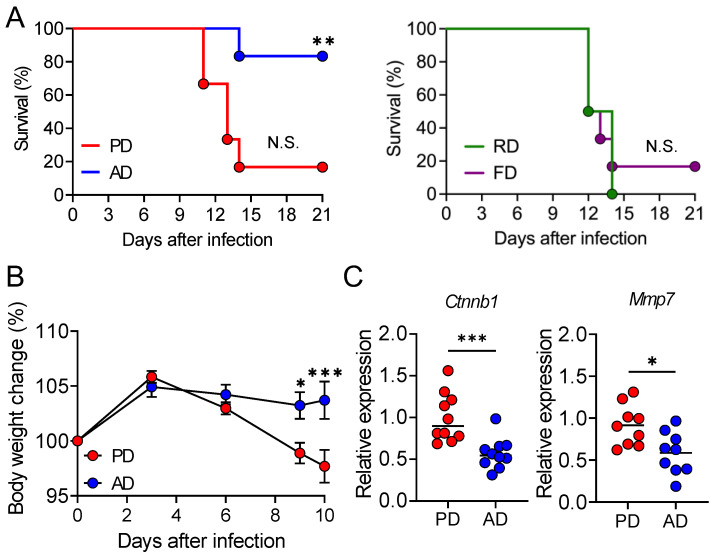
An amino acid-based diet protects mice from lethal infection with *Citrobacter rodentium*. Mice fed each diet (RD: regular natural diet (*n* = 10), PD: regular purified diet (*n* = 10), AD: amino acid-based purified diet (*n* = 10), FD: purified high-fat diet (*n* = 10)) were orally administered *C. rodentium*. (**A**) Mouse survival and (**B**) body weight change 3, 6, 9, and 10 days post-infection compared to that on day 0. Data are expressed as mean ± SEM. (**C**) Gene expression of *Ctnnb1* and *Mmp7* in the colon 9 days post-infection by real-time RT-PCR. mRNA expression of each gene was normalized to that of *Rpl19*. Each dot represents an individual mouse, and horizontal bars indicate mean values. Statistical significance was assessed by the (**A**) log-rank test, compared to RD group, (**B**) two-way ANOVA with Šídák’s multiple comparisons test, or (**C**) unpaired Student’s *t*-test. * *p* < 0.05; ** *p* < 0.01; *** *p* < 0.001; N.S., not significant.

To assess the impact of diets on lethal infectious diarrhea, AD, FD, PD, and RD-fed mice were infected with *C. rodentium,* and their survival was monitored over time. More than 90% of RD, PD, and FD-fed mice succumbed to infection, compared to only 20% of AD-fed mice (Figure 1A). Since only the protein sources of the PD and AD differed (casein vs. amino acids), we next focused on PD and AD-fed mice. Body weight loss was significantly greater in PD-fed mice than in AD-fed mice throughout the duration of infection (Figure 1B). Wnt signaling activation is one of the hallmarks of diarrheal *C. rodentium* infection susceptibility [37]. Therefore, we compared the expressions of β-catenin (*Ctnnb1*), the principal canonical Wnt signaling downstream mediator, and MMP-7 (*Mmp7*), which is transcriptionally regulated by β-catenin. The expression of Wnt target genes *Ctnnb1* and *Mmp7* was significantly lower in the AD-fed mice colon than that in PD-fed mice colon (Figure 1C). These results indicate that an AD protects mice from lethal *C. rodentium* infection.

### 3.2. AD neither Promotes Pathogen Clearance nor Suppresses Intestinal Damage

**Figure 2:**
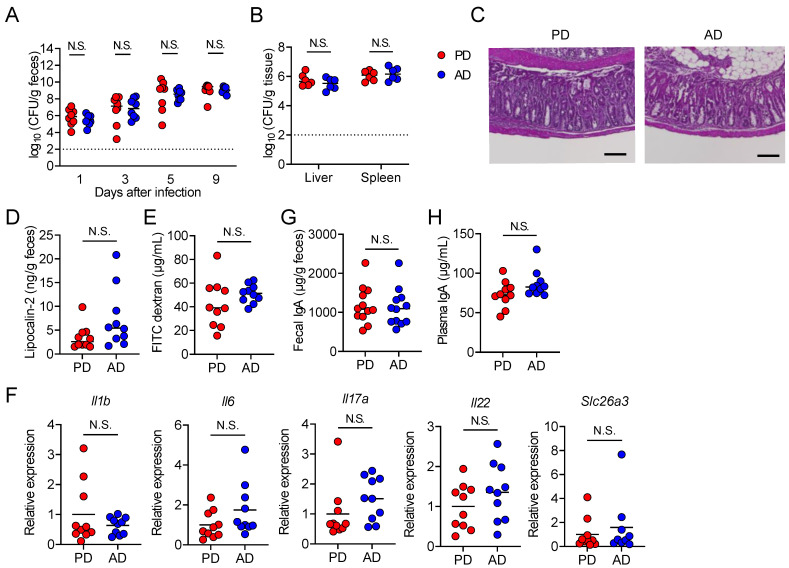
An amino acid-based diet neither promotes pathogen clearance nor suppresses intestinal damage. (**A**–**F**) Mice fed each diet (PD; regular purified diet, AD; amino acid-based purified diet) were orally infected with *C. rodentium* (*n* = 9–10 in each group). (**A**,**B**) Fecal pathogen load on 1, 3, 5, and 9 days post-infection (**A**) and in the tissues 10 days post-infection (**B**). (**C**) H&E staining of colon slides from representative infected mice fed PD or AD 10 days post-infection. scale bar, 200 μm (**D**) Fecal lipocalin-2 concentration 9 days post-infection. (**E**) Plasma concentration of fluorescein isothiocyanate (FITC)-dextran 7 days post-infection. (**F**) Gene expression of *Il1b*, *Il6*, *Il17a*, *Il22*, and *Slc26a3* in the colon 9 days post-infection by real-time RT-PCR. mRNA expression of each gene was normalized to that of *Rpl19* (**G**–**H**) Mice fed PD or AD for 2 weeks (*n* = 10 in each group). IgA levels in (**G**) feces or (**H**) plasma. Each dot represents an individual mouse, and the horizontal bars indicate mean values. Statistical significance was assessed by two-way ANOVA with (**A**,**B**) Šídák’s multiple comparisons test, (**D**,**F** (*Il1b*, *Il6*, *Il17a*, *Slc26a3*), **H**) Mann–Whitney test, (**E**,**F** (*Il22*)) unpaired Welch’s *t*-test, or (**G**) unpaired Student’s *t*-test. N.S., not significant.

Next, we examined whether an AD influences pathogen clearance and gut barrier function. Fecal *C. rodentium* shedding was detected as early as 1 day post-infection and was not significantly different between PD and AD-fed mice 9 days post-infection (Figure 2A). The bacterial burden in the liver and spleen of PD and AD-fed mice was also similar (Figure 2B). After the *C. rodentium* infection, severe epithelial hyperplasia and a loss of crypt morphology were observed, but the histological changes of the colon were not apparently different between the mice fed a PD and AD (Figure 2C). Consistently, the fecal level of lipocalin-2, an inflammatory marker, was comparable in both groups 9 days post-infection (Figure 2D). We then assessed the intestinal barrier function in PD and AD-fed mice by measuring the plasma level of the orally administered permeability marker FITC-dextran. The plasma level of FITC-dextran was not different between the groups 7 days post-infection (Figure 2E). In addition, the colonic expression of inflammatory cytokines, *Il1b*, *Il6*, *Il17a*, *Il22*, and *Slc26a3*, which encode the major Cl^−^/HCO_3_^−^ exchanger that drives water absorption [38], was comparable between the infected mice fed a PD and AD (Figure 2F). Furthermore, the levels of fecal (intestinal) IgA, which prevents enteric pathogen invasion [39], and plasma IgA, which protects against polymicrobial sepsis [40], were not significantly different between PD and AD-fed mice prior to the infection (Figure 2G,H). Collectively, we concluded that an AD neither promotes pathogen elimination nor suppresses *C. rodentium*-induced intestinal damage, inflammation, and sepsis.

### 3.3. AD Promotes Water Intake, Thereby Protecting from Lethal Infectious Diarrhea

**Figure 3:**
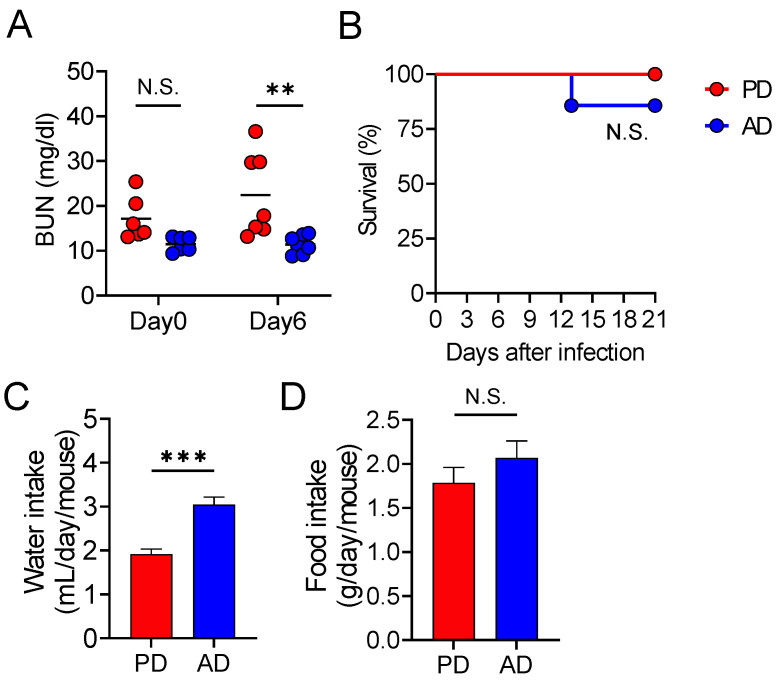
An amino acid-based diet promotes water intake and protects from lethal infectious diarrhea. (**A**,**B**) Mice fed each diet (PD; regular purified diet, AD; amino acid-based purified diet) were infected orally with *Citrobacter rodentium* (*n* = 9–10 in each group). (**A**) Blood urea nitrogen (BUN) level. Each dot represents an individual mouse and the horizontal bars indicate mean values. (**B**) Mouse survival after peritoneal saline treatment once a day after 6 days. (**C**,**D**) Mice fed PD or AD for 2 weeks (*n* = 9–10 in each group). (**C**) Water and (**D**) food intake. Data are expressed as mean ± SD. Statistical significance was assessed by two-way ANOVA with (**A**) Šídák’s multiple comparisons test, (**B**) log-rank test, (**C**) unpaired Student’s *t*-test, or (**D**) Mann–Whitney test. ** *p* < 0.01; *** *p* < 0.001; N.S., not significant.

Mortality in *C. rodentium*-infected mice is associated with fatal fluid loss and dehydration [16]. Therefore, we assessed whether a PD prevents *C. rodentium* infection-induced dehydration. We found that the level of blood urea nitrogen (BUN), a dehydration status marker, was significantly lower in AD-fed mice than that in PD-fed mice (Figure 3A). Moreover, peritoneal saline treatment protected the PD-fed mice from *C. rodentium* infection, and the survival rate was comparable to that of AD-fed mice (Figure 3B). Notably, AD-fed mice had a higher water intake than PD-fed mice, although the amount of food consumed was not different (Figure 3C,D). These results indicate that an AD promotes water intake, which protects the mice from *C. rodentium* infection-induced dehydration and death.

### 3.4. AD Changes Plasma and Fecal Amino Acid Levels

**Figure 4:**
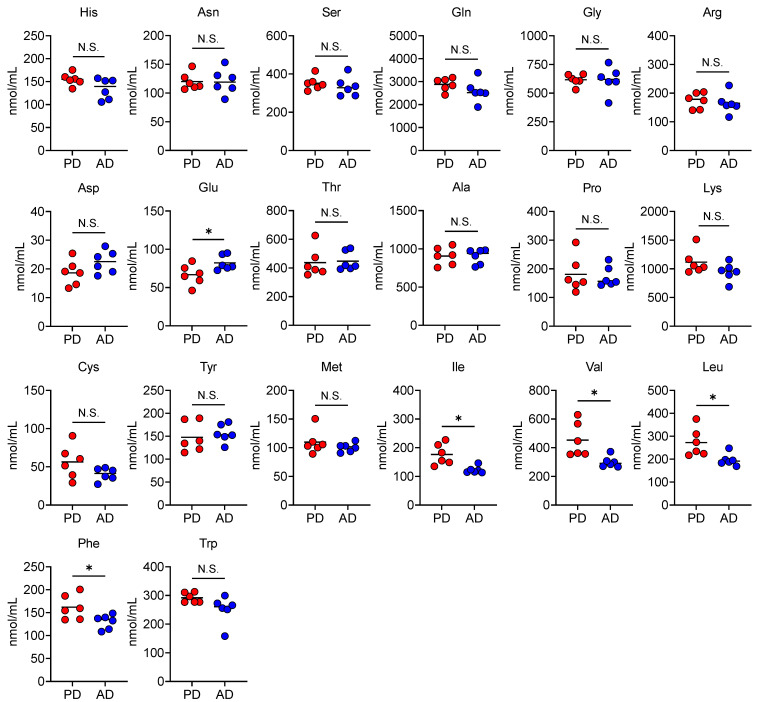
An amino acid-based diet alters plasma amino acid levels. Plasma levels of each amino acid in the mice fed each diet (PD: regular purified diet, AD: amino acid-based purified diet) for 2 weeks (*n* = 6 in each group). Each dot represents an individual mouse, and horizontal bars indicate mean values. Statistical significance was assessed using the unpaired Welch’s *t*-test. * *p* < 0.05; N.S., not significant.

**Figure 5:**
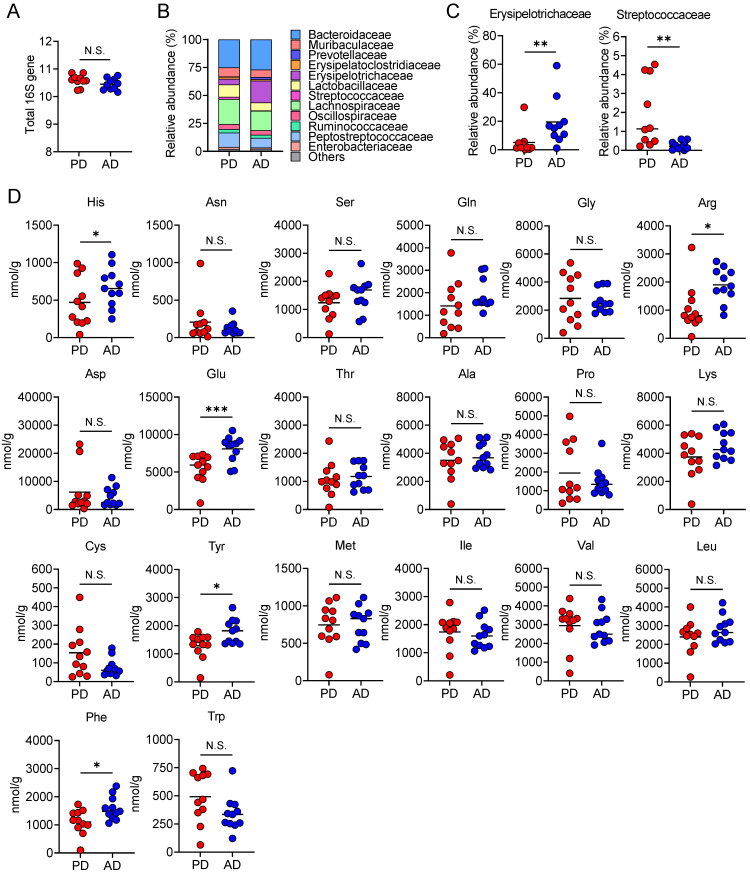
An amino acid-based diet changes gut microbiota composition and fecal amino acid levels. Mice fed each diet (PD, regular purified diet; AD, amino acid-based purified diet) for 2 weeks (*n* = 6 in each group). (**A**) Total bacterial number in the feces. (**B**) Relative abundance of operational taxonomic units (OTUs) in fecal samples from each group. The various colors correspond to each bacterial order. (**C**) Relative abundance of OTUs assigned to Erysipelotrichaceae and Streptococcaceae families. (**D**) Fecal levels of each amino acid. Each dot represents an individual mouse, and horizontal bars indicate mean values. Statistical significance was assessed by (**A**) unpaired Student’s *t*-test, (**C**) Mann–Whitney test, or (**D**) unpaired Welch’s *t*-test. * *p* < 0.05; ** *p* < 0.01; *** *p* < 0.001; N.S., not significant.

Dietary protein composition and plasma amino acid concentration are associated with water intake [41,42]. Therefore, we compared plasma amino acid concentrations between PD and AD-fed mice. Plasma glutamate levels were higher and the levels of isoleucine, leucine, valine, and phenylalanine were lower in the AD-fed mice than those in the PD-fed mice (Figure 4). Dietary protein composition is one of the primary factors contributing to the composition, structure, and function of gut microbes, which can in turn provide amino acids to the host [43,44]. Thus, we examined whether an AD influences the composition of the gut microbial communities. Although it did not affect the total bacterial number (Figure 5A), an AD changed the gut microbiota composition (Figure 5B) by significantly increasing and decreasing the abundance of Erysipelotrichaceae and Streptococcaceae families, respectively (Figure 5C). Furthermore, the fecal histidine, arginine, glutamate, tyrosine, and phenylalanine concentrations were higher in AD-fed mice than in PD-fed mice (Figure 5D). Collectively, these results indicate that an AD alters plasma and fecal amino acid levels, which may maintain the total body water content, thereby protecting the mice from lethal diarrheal *C. rodentium* infection.

### 3.5. Oral Glutamate Treatment Protects Mice from Lethal Diarrheal C. rodentium Infection

**Figure 6:**
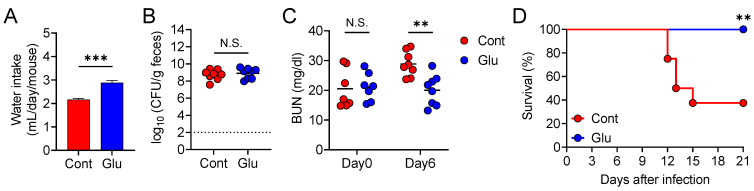
Oral glutamate treatment protects mice from lethal diarrheal *Citrobacter rodentium* infection. (**A**) Mice were treated with or without 1% glutamate for 10 days. Water intake of each group. Data are expressed as mean ± SD. (**B**–**D**) Mice treated with or without 1% glutamate were infected orally with *C. rodentium* (*n* = 8 in each group). (**B**) Fecal pathogen load 6 days post-infection. (**C**) Blood urea nitrogen (BUN) level 0 and 6 days post-infection. (**D**) Mouse survival. Each dot represents an individual mouse and the horizontal bars indicate mean values. Statistical significance was assessed by (**A**,**B**) unpaired Student’s *t*-test or (**C**) two-way ANOVA with Šídák’s multiple comparisons test. ** *p* < 0.01; *** *p* < 0.001; N.S., not significant.

Since an AD increased both plasma and fecal glutamate levels, we hypothesized that glutamate influences water intake and protects the mice from diarrheal *C. rodentium* infection-induced dehydration. Therefore, we assessed whether glutamate treatment prevents *C. rodentium* infection-induced lethal diarrhea. Water intake increased in glutamate-treated mice compared to that in the untreated mice (Figure 6A). Although glutamate treatment did not suppress *C. rodentium* colonization (Figure 6B), it prevented *C. rodentium*-induced dehydration, as assessed by BUN levels (Figure 6C). Consistently, glutamate administration significantly improved the survival of *C. rodentium*-infected mice (Figure 6D). These results indicate that glutamate treatment promotes water intake, prevents diarrheal *C. rodentium* infection-induced dehydration, and improves mouse survival.

## 4. Discussion

Murine *C. rodentium* infection is an excellent model for studying important disease processes, including infectious diarrhea. This infection model is characterized by colonic hyperplasia, inflammation, and diarrhea, depending on the age, diet, microbiological status, and genetic background of the host. Using the *C. rodentium* infection model, we have shown that an AD protects mice from fatal infectious diarrhea by promoting water intake.

A previous report has shown that chronic monosodium glutamate administration increases water intake [45]. In addition, glutamine stimulates water and sodium absorption in enterocytes [46]. Oral glutamate treatment also increased water intake, prevented dehydration, and improved the survival of *C. rodentium*-infected mice. Therefore, an AD may protect against *C. rodentium* infection-induced dehydration, partly by increasing the plasma and intestinal glutamate levels. We used a glutamic acid sodium salt for oral glutamate treatment. Water ionizes sodium glutamate into free sodium ions and glutamic acids. Therefore, sodium may increase water intake. However, sodium solution intake was decreased in C3H mice compared to water intake [47]. Thus, the increased water intake was caused by glutamic acid, and not sodium. In contrast, the plasma concentrations of branched-chain amino acids (BCAAs; isoleucine, leucine, and valine) were higher in PD-fed mice than those in AD-fed mice. The BCAA-enriched diet reduced water intake [48]. Furthermore, severe dehydration due to gastroenteritis often causes metabolic acidosis, which increases plasma BCAA concentrations due to the hypermetabolic states of proteolysis [49]. Therefore, increased plasma BCAAs may be detrimental to diarrhea-related dehydration.

Wnt/β-catenin signaling promotes intestinal epithelial proliferation and the generation of a poorly differentiated epithelium, leading to the downregulation of ion transporters such as Slc26a3, which is exclusively expressed in differentiated enterocytes, thereby reducing water absorption and inducing dehydration [37]. The expression of *Ctnnb1* and *Mmp7* was significantly lower in the colon of the mice fed an AD than the mice fed a PD, suggesting that the activation of Wnt signaling pathways is suppressed by an AD. However, the expression of *Slc26a3* was comparable in the infected mice fed a PD and AD. Thus, an AD may protect the mice from lethal infection diarrhea by promoting water intake rather than improving water absorption. Further work will reveal the mechanism by which an AD promotes water intake.

An AD increased the fecal concentration of several amino acids. The distribution of free amino acids in the intestine, brain, and serum of germ-free mice was different from that in conventionalized mice [50,51], suggesting that gut microbiota contributes to host amino acid balance. Clostridia, Peptostreptococci, *Bacillus*–*Lactobacillus*–*Streptococcus* groups, and Proteobacteria are the most prevalent species involved in amino acid fermentation in the human intestine [52,53,54]. Such bacteria are likely to influence the amino acid pools in the gut. Accordingly, the abundance of Streptococcaceae, which contains *Streptococcus*, decreased in the gut of AD-fed mice. Therefore, changes in gut microbiota composition due to an AD may also affect fecal and plasma amino acid levels. An AD increased the abundance of Erysipelotrichaceae, which belongs to *Clostridium* Cluster XVI, which includes Gram-positive filamentous rods and both facultative and obligate anaerobes. High-protein diets increase Erysipelotrichaceae abundance and upregulate the genes involved in amino acid metabolism and transport, as well as proteolysis [55]. Therefore, an AD may supply the free amino acids utilized by Erysipelotrichaceae for growth.

In conclusion, we observed that an AD prevents fatal infectious diarrhea by regulating body water balance. Our findings provide new insight into current oral rehydration therapy by providing glucose and electrolyte solutions to prevent and/or correct diarrhea-induced dehydration.

## Figures and Tables

**Table 1:** Nutrient composition of experimental diets.

Diet	PD	AD	FD	RD *
	g%	g%	g%	g%
Protein	20	18	26.2	24.8 **
Carbohydrate	64	66	26.3	54.55 ***
Fat	7	7	34.9	4.6 ****
(kcal/gm)	4	4	5.2	3.4
**Ingredient**	**g**	**g**	**g**	**g**
Casein	200	0	258.4	-
L-Cystine	3	4.3	3.9	-
L-Arginine	-	6.1	-	-
L-Histidine-HCl-H_2_O	-	4.7	-	-
L-Isoleucine	-	7.7	-	-
L-Leucine	-	16.0	-	-
L-Lysine-HCl	-	13.4	-	-
L-Methionine	-	5.2	-	-
L-Phenylalanine	-	8.5	-	-
L-Threonine	-	7.3	-	-
L-Tryptophan	-	2.1	-	-
L-Valine	-	9.4	-	-
L-Alanine	-	5.2	-	-
L-Asparagine-H_2_O	-	6.8	-	-
L-Aspartate	-	5.5	-	-
L-Glutamic Acid	-	22.0	-	-
L-Glutamine	-	16.7	-	-
Glycine	-	3	-	-
L-Proline	-	18.1	-	-
L-Serine	-	10.1	-	-
L-Tyrosine	-	9.3	-	-
Corn Starch	397.5	403.4	0	-
Maltodextrin	132	134	162	-
Sucrose	100	108.7	88.9	-
Cellulose, BW200	50	51	65	-
Corn Oil	0	0	0	-
Soybean Oil	70	71	32	-
Lard	0	0	317	-
t-butylhydroquinone	0	0	0	-
**Vitamins**	**mg**	**mg**	**mg**	**mg**
Vitamin A	6.6	6.7	8.5	8.1
Vitamin B1	5.3	5.4	6.8	18
Vitamin B2	6	6.1	7.8	14
Vitamin B6	5.8	5.9	7.5	13
Vitamin B12	2.5	2.5	1.3	0
Vitamin D3	10	10.1	12.9	0.1
Vitamin E	131.7	133.7	113.5	71
Pantothenic acid	13.5	13.7	17.4	30
Biotin	20	2	2.6	0.5
Folic Acid	2	6.7	8.5	8.1
Total (g)	1000	1000	1000	1000

* RD; regular natural diet, PD; regular purified diet, AD; amino acid-based purified diet, FD; purified high-fat diet. Average of periodic analysis in 2020. ** Protein source: soybean waste, whitefish meal, yeast. *** Carbohydrate source: wheat flour, corn, Milo (fiber source: wheat bran, defatted rice bran, alfalfa meal). **** Fat source: cereal germ, soybean oil. All diets contain appropriate minerals.

**Table 2:** Primer sequences.

Genes		Primer Sequence
*Ctnnb1*	Forward	ATG GAG CCG GAC AGA AAA GC
	Reverse	TGG GAG GTG TCA ACA TCT TCT T
*Mmp7*	Forward	GCA TTT CCT TGA GGT TGT CC
	Reverse	CAC ATC AGT GGG AAC AGG C
*Il1b*	Forward	GAA ATG CCA CCT TTT GAC AGT G
	Reverse	TGG ATG CTC TCA TCA GGA CAG
*Il6*	Forward	TGA TGC ACT TGC AGA AAA CA
	Reverse	ACC AGA GGA AAT TTT CAA TAG GC
*Il17a*	Forward	TCAGCGTGTCCAAACACTGAG
	Reverse	CGCCAAGGGAGTTAAAGACTT
*Il22*	Forward	GTG CTC AAC TTC ACC CTG GA
	Reverse	TGG ATG TTC TGG TCG TCA CC
*Slc26a3*	Forward	AACATCCCTCCAGCCTACG
	Reverse	TGGACCCACAGATATGTGTCT
*Rpl19*	Forward	TAC CGG GAA TCC AAG AAG ATT GA
	Reverse	AGG ATG CGC TTG TTT TTG AAC

## Data Availability

The data presented in this study are available on request from the corresponding author.
